# Evolution of Respiratory Pathogens and Antimicrobial Resistance over the COVID-19 Timeline: A Study of Hospitalized and Ambulatory Patient Populations

**DOI:** 10.3390/antibiotics14080796

**Published:** 2025-08-05

**Authors:** Luigi Regenburgh De La Motte, Loredana Deflorio, Erika Stefano, Matteo Covi, Angela Uslenghi, Carmen Sommese, Lorenzo Drago

**Affiliations:** 1 UOC Laboratory of Clinical Medicine with Specialized Areas, IRCCS MultiMedica, 20138 Milan, Italy; luigi.delamotte@multimedica.it (L.R.D.L.M.); loredana.deflorio@multimedica.it (L.D.); erika.stefano@multimedica.it (E.S.); matteo.covi@multimedica.it (M.C.); angela.uslenghi@multimedica.it (A.U.); 2 Lab of Cardiovascular Diabetology and Dysmetabolic Disease, MultiMedica Scientific Institute for Hospitalization and Care (IRCCS), 20138 Milan, Italy; carmen.sommese@multimedica.it; 3 Clinical Microbiology and Microbiome Laboratory, Department of Biomedical Sciences for Health, University of Milan, 20133 Milan, Italy

**Keywords:** respiratory infections, antimicrobial resistance, COVID-19 pandemic, bacterial epidemiology, polymicrobial colonization

## Abstract

**Background:** The COVID-19 pandemic has profoundly altered the clinical and microbiological landscape of respiratory tract infections (RTIs), potentially reshaping pathogen distribution and antimicrobial resistance (AMR) profiles across care settings. **Objectives:** The objective of this study was to assess temporal trends in respiratory bacterial pathogens, antimicrobial resistance, and polymicrobial infections across three pandemic phases—pre-COVID (2018–2019), COVID (2020–2022), and post-COVID (2022–2024)—in hospitalized and ambulatory patients. **Methods:** We retrospectively analyzed 1827 respiratory bacterial isolates (hospitalized patients, *n* = 1032; ambulatory patients, *n* = 795) collected at a tertiary care center in Northern Italy. Data were stratified by care setting, anatomical site, and pandemic phase. Species identification and susceptibility testing followed EUCAST guidelines. Statistical analysis included chi-square and Fisher’s exact tests. **Results:** In hospitalized patients, a significant increase in *Pseudomonas aeruginosa* (from 45.5% pre-COVID to 58.6% post-COVID, *p* < 0.0001) and *Acinetobacter baumannii* (from 1.2% to 11.1% during COVID, *p* < 0.0001) was observed, with 100% extensively drug-resistant (XDR) rates for *A. baumannii* during the pandemic. Conversely, *Staphylococcus aureus* significantly declined from 23.6% pre-COVID to 13.7% post-COVID (*p* = 0.0012). In ambulatory patients, polymicrobial infections peaked at 41.2% during COVID, frequently involving co-isolation of *Candida* spp. Notably, resistance to benzylpenicillin in *Streptococcus pneumoniae* reached 80% (4/5 isolates) in hospitalized patients during COVID, and carbapenem-resistant *P. aeruginosa* (CRPA) significantly increased post-pandemic in ambulatory patients (0% pre-COVID vs. 23.5% post-COVID, *p* = 0.0014). **Conclusions:** The pandemic markedly shifted respiratory pathogen dynamics and resistance profiles, with distinct trends observed in hospital and community settings. Persistent resistance phenotypes and frequent polymicrobial infections, particularly involving *Candida* spp. in outpatients, underscore the need for targeted surveillance and antimicrobial stewardship strategies.

## 1. Introduction

Respiratory tract infections (RTIs) represent a significant global health concern, contributing to high morbidity and mortality rates, particularly among vulnerable populations, such as hospitalized, immunocompromised, or elderly patients. These infections encompass a wide spectrum of clinical conditions, from mild upper respiratory tract infections to severe pneumonia and sepsis, and are caused by a diverse group of pathogens, including *Pseudomonas aeruginosa*, *Staphylococcus aureus*, *Klebsiella pneumoniae*, and *Streptococcus pneumoniae* [1,2,3]. The clinical management of RTIs is further complicated by the rising burden of antimicrobial resistance (AMR), with the emergence of multidrug-resistant (MDR) organisms limiting therapeutic options and adversely affecting outcomes [4,5].

The COVID-19 pandemic has profoundly altered the landscape of respiratory infections. While the widespread implementation of public health measures such as lockdowns, mask mandates, and social distancing led to a marked reduction in community-acquired respiratory infections—particularly viral pathogens such as influenza and respiratory syncytial virus—concomitant shifts in healthcare delivery and antibiotic prescribing practices have impacted the epidemiology of bacterial RTIs [6,7,8]. Multiple studies have reported a paradoxical increase in healthcare-associated infections (HAIs), including ventilator-associated pneumonia (VAP), driven by ICU overcrowding, increased use of invasive procedures, and empiric broad-spectrum antibiotic therapy [5,6,8].

In Italy, national surveillance data from the Istituto Superiore di Sanità (ISS) and the European Centre for Disease Prevention and Control (ECDC) have documented concerning increases in carbapenem-resistant *K. pneumoniae* and *P. aeruginosa*, as well as in penicillin- and macrolide-resistant *S. pneumoniae* [2,4,5,9]. These trends underscore the dual challenge posed by the pandemic: managing the immediate threat of SARS-CoV-2 while containing secondary bacterial infections, which can exacerbate disease severity and overwhelm healthcare systems [5,7,8].

Among bacterial pathogens, *S. pneumoniae* holds particular importance due to its role as a leading cause of community-acquired pneumonia and its value as a sentinel organism in AMR surveillance. The resistance to beta-lactams—particularly benzylpenicillin—has epidemiological significance across Europe and is monitored to guide empirical therapy [2]. Furthermore, polymicrobial respiratory infections—often overlooked—are increasingly recognized for their role in complicating clinical trajectories, especially in outpatient settings and immunocompromised individuals [10,11].

The frequent isolation of *Candida* spp. in respiratory specimens, while traditionally regarded as colonization, has been proposed as a surrogate marker of dysbiosis and excessive antibiotic use [12,13,14,15]. Although its clinical role in RTIs remains debated, its presence in polymicrobial infections may serve as an indirect indicator of antimicrobial pressure.

This study aims to provide a comprehensive assessment of bacterial respiratory infections and AMR patterns across three epidemiologically distinct periods: pre-COVID (June 2018–December 2019), COVID (January 2020–July 2022), and post-COVID (August 2022–June 2024). By stratifying data by patient setting (inpatients vs. outpatients), anatomical site (upper vs. lower respiratory tract), and pandemic phase, we aim to elucidate temporal shifts in pathogen prevalence, resistance phenotypes, and the occurrence of polymicrobial infections. Our study specifically examined resistance patterns of WHO priority pathogens such as methicillin-resistant *S. aureus* (MRSA), extended-spectrum beta-lactamase (ESBL)-producing *K. pneumoniae*, and carbapenem-resistant *Enterobacterales* (CRE), highlighting their epidemiological significance in the context of COVID-19 pandemic dynamics, as well as the epidemiological relevance of *Candida* spp. and polymicrobial respiratory profiles. Through this lens, this study contributes to a better understanding of how the COVID-19 pandemic has shaped the respiratory microbiome and informs future infection control and antimicrobial stewardship strategies.

## 2. Materials and Methods

### 2.1. Patients

This retrospective observational study was conducted at the Laboratory of Clinical Medicine with Specialized Areas, IRCCS MultiMedica, and analyzed microbiological data from lower and upper respiratory tract samples collected from both hospitalized and ambulatory patients. The samples were collected at the IRCCS MultiMedica hospitals (Sesto San Giovanni Hospital and San Giuseppe Hospital), located in Milan, Northern Italy. As a reference laboratory, we received and processed respiratory specimens for culture analysis and antibiotic susceptibility testing. All routine clinical respiratory samples from patients presenting signs or symptoms of respiratory tract infections were included. Duplicate isolates from the same patient within the same infection episode were excluded.

### 2.2. Data Collection

Patient demographic data (age, sex, hospitalization status) and microbiological results were retrieved using the hospital’s electronic medical record system as the primary data collection tool. The study was conducted using anonymized bacterial isolates derived from different matrix collected during routine clinical practice in patients with RTIs, following the Ethical Committee approval (Prot. Nr. 378/24, Comitato Etico Territoriale Lombardia, Italy) on 23 July 2024. All residual clinical respiratory samples were accessed for research purposes between 1 July 2018 and 30 June 2024. Furthermore, the study was conducted in accordance with the Declaration of Helsinki. Data were stratified across three epidemiological timeframes (Table 1):

- Pre-COVID: June 2018–December 2019;
- COVID: January 2020–July 2022;
- Post-COVID: August 2022–June 2024.

During these periods, a total of 1827 bacterial isolates were identified.

Samples were categorized as lower respiratory tract specimens (bronchoalveolar lavage [BAL], bronchoaspirate [BAS], and sputum [ESP]) or upper respiratory tract specimens (nasal/pharyngeal swabs [TNAS, and TAU], and pharyngeal swabs [COLTFAR]). Each infection was classified as monomicrobial or polymicrobial based on culture results. Polymicrobial infections were defined as the isolation of two or more clinically significant bacterial species from the same respiratory sample.

Antimicrobial susceptibility testing was performed on viable isolates, enabling an evaluation of resistance trends across the three pandemic periods.

### 2.3. Microbiological Analysis

All respiratory samples were processed at the MultiMedica Laboratory. Microbiological identification and antimicrobial susceptibility testing followed standard quality assurance protocols as indicated by AMCLI and EUCAST guidelines, ensuring data accuracy and reliability (https://www.amcli.it/ (accessed on 10 June 2025)). Bacterial identification and susceptibility testing were performed using culture-based methods and the VITEK^®^ 2 automated system (bioMérieux), following EUCAST interpretive criteria (https://www.eucast.org/ (accessed on 10 June 2025)).

Among the 1827 isolates, the analysis focused on the most prevalent and clinically relevant bacterial species identified in all three study periods. These organisms were selected based on their frequency, role in respiratory infections, and relevance for hospital-associated infections and antimicrobial resistance (AMR) surveillance. To ensure robustness, only species with ≥5 isolates across the entire period were included in resistance trend analyses. Bacteria isolated fewer than 10 times overall were excluded from statistical comparisons but are listed in the Supplementary Material; the tables also include the antimicrobial resistance (Tables S1–S3).

Resistance mechanisms were classified according to ECDC and WHO definitions, including the following:

Methicillin-resistant *Staphylococcus aureus* (MRSA);

Extensively drug-resistant *Acinetobacter baumannii* (XDR-AB);

Carbapenem-resistant *Pseudomonas aeruginosa* (CRE);

Extended-spectrum β-lactamase-producing (ESBL+) and carbapenem-resistant (CRE) *Klebsiella pneumoniae*;

Benzylpenicillin and macrolide resistance in *Streptococcus pneumonia*;

Macrolide resistance in *Streptococcus pyogenes*.

The dataset included detailed metadata for each isolate, including collection date, microbial identification, antibiotic susceptibility profiles, and classification of resistance.

### 2.4. Statistical Analysis

To evaluate bacterial prevalence across the three study periods, the prevalence of polymicrobial infections, and antimicrobial resistance (AMR) trends, statistical analyses were performed. The Chi-square test for independence was applied to compare bacterial prevalence, AMR rates, and setting-based differences (hospitalized vs. outpatient) over time, while Fisher’s exact test was used when expected frequencies were low. To assess differences in the prevalence of polymicrobial infections between the pre-COVID and post-COVID periods, both the Chi-square and Fisher’s exact tests were employed, as appropriate. A multivariate logistic regression model was constructed to identify independent predictors of resistance, adjusting for time period and bacterial species. Statistical significance was defined as *p* < 0.05. All analyses were conducted using GraphPad Prism version 8.0.1 (GraphPad Software, San Diego, CA, USA).

### 2.5. Study Objectives

The primary objective of this study was to investigate temporal trends in bacterial prevalence, the rate of polymicrobial infections, and the evolution of antimicrobial resistance in patients with respiratory infections across three key phases of the COVID-19 pandemic. This approach aimed to elucidate the dynamic interplay between pathogen ecology, co-infection complexity, and resistance evolution in a pandemic-influenced healthcare environment.

## 3. Results

### 3.1. Microbiological Distribution: Inpatients vs. Outpatients

A total of 1827 bacterial respiratory isolates were collected across the three study periods: 593 in the pre-COVID phase (June 2018–December 2019), 796 during COVID (January 2020–July 2022), and 438 in the post-COVID phase (August 2022–June 2024). Among these, 1032 isolates (56.5%) were from hospitalized patients and 795 (43.5%) from ambulatory patients (Table 2 and Table 3, Figure S2).

In hospitalized patients, *P. aeruginosa* was the most prevalent pathogen across all timeframes, increasing significantly from 45.5% in the pre-COVID period to 46.8% during COVID, and further to 58.6% post-COVID (*p* < 0.0001). *K. pneumoniae* remained relatively stable, with values of 18.3%, 18.9%, and 15.2% across the respective periods (*p* = 0.4639). A notable surge was observed in *A. baumannii* complex during the pandemic peak (from 1.2% to 11.1%), followed by a sharp decline post-pandemic (3.1%), with statistical significance (*p* < 0.0001). In contrast, *S. aureus* declined markedly from 23.6% pre-COVID to 20.4% during COVID and 13.7% post-COVID (*p* = 0.0012), while *S. pneumoniae* showed no significant fluctuations over time (1.6%, 0.9%, and 1.6%; *p* = 0.7655).

In ambulatory patients, the most striking trend was the consistent presence of *Candida* spp., accounting for 30.3% of isolates pre-COVID, 33.1% during COVID, and 26.9% post-COVID, without statistically significant variation (*p* = 0.3780). *S. pyogenes*, exclusive to this population, showed a significant rise from 10.7% pre-COVID to 14.8% post-COVID (*p* = 0.0004). Conversely, *S. aureus* declined sharply from 24.2% to 7.1% (*p* < 0.0001), while *P. aeruginosa* significantly increased from 14.4% to 34.6% across the three periods (*p* < 0.0001). The prevalence of *K. pneumoniae* and *S. pneumoniae* remained low and stable, with no significant temporal variation.

These findings underscore a pandemic-driven shift in the respiratory microbiological landscape, with *P. aeruginosa* and *Candida* spp. emerging as dominant organisms in hospitalized and outpatient settings, respectively. The observed rise in multidrug-resistant Gram-negative bacteria, along with a concurrent decline in typical Gram-positive pathogens such as *S. aureus* and *S. pneumoniae* points to evolving ecological dynamics in both inpatient and ambulatory contexts. Notably, the exclusive isolation of *Candida* spp. in outpatient samples and the consistent presence of *S. pyogenes* only in the ambulatory cohort merit further exploration.

### 3.2. Polymicrobial Infections

In ambulatory patients, polymicrobial infections were more frequently observed in lower respiratory tract samples, with the highest rate during the COVID period (42/102, 41.2%), followed by the pre-COVID (34/87, 39.1%) and post-COVID (17/62, 27.4%) periods. A consistent feature across all timeframes was the frequent co-isolation of *Candida spp*., particularly in bronchoaspirate and sputum samples. This trend suggests a stable pattern of fungal–bacterial co-infections in the outpatient population, possibly influenced by underlying comorbidities or environmental exposures.

Sputum samples also exhibited notable rates of polymicrobial infections, reaching 32.8% in the pre-COVID period and 30.4% during the pandemic. Although this decreased post-COVID (17.4%), *Candida* spp. remained one of the most common co-isolated organisms. In contrast, pharyngeal and nasal swabs showed fewer polymicrobial patterns overall, and a lower frequency of fungal detection.

**Figure 1:**
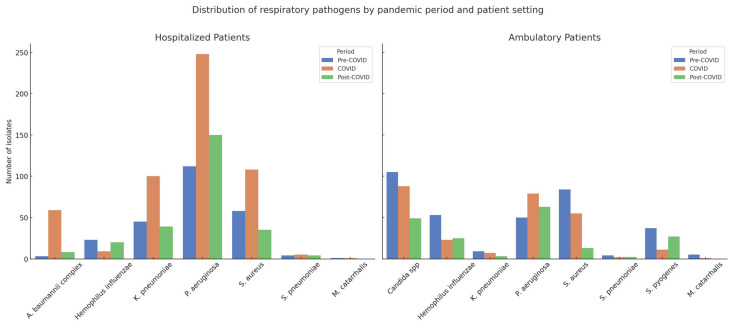
Distribution of different bacterial isolates from hospitalized and ambulatory patients during the three periods.

Interestingly, *Candida* spp. was not detected in hospitalized patients, where polymicrobial infections instead involved opportunistic Gram-negative pathogens such as *P. aeruginosa* and *A. baumannii* complex. This clear setting-dependent dichotomy underscores the differing clinical and ecological pressures shaping respiratory microbiota in ambulatory versus inpatient care (Figure 1 and Figure S1).

### 3.3. Antimicrobial Resistance Patterns

Antimicrobial resistance (AMR) patterns exhibited substantial fluctuations across the three epidemiological periods, with distinct profiles observed in hospitalized and ambulatory patient populations.

#### 3.3.1. Hospitalized Patients

*P. aeruginosa*: Resistance to carbapenems (CRE) significantly decreased from 32.1% (36/112) in the pre-COVID period to 20.2% (50/248) during COVID (*p* = 0.0163) and further declined to 16.7% (25/150) post-COVID. This trend may reflect improved antimicrobial stewardship and infection control measures during the pandemic peak.

*S. aureus* (MRSA): The absolute number of MRSA cases increased from 35 pre-COVID to 62 during COVID, before dropping to 21 post-COVID. However, when normalized to total isolates, the prevalence remained relatively stable, and changes were not statistically significant.

*K. pneumoniae* (ESBL+): The proportion of ESBL-producing isolates rose from 33.3% (15/45) pre-COVID to 27.0% (27/100) during COVID, then declined to 17.9% (7/39) post-COVID. Although the absolute number peaked during the pandemic, the trend was not statistically significant (*p* > 0.05).

*A. baumannii* complex (XDR-AB): A pronounced increase in extensively drug-resistant isolates was observed during COVID (from 1.2% to 11.1%), dropping to 3.1% post-pandemic. Despite the sharp fluctuation, statistical significance was not reached.

*S. pneumoniae*: Resistance to benzylpenicillin increased to 80.0% (4/5) during the COVID period in hospitalized patients. Although the total number of isolates was low, this spike holds epidemiological significance, particularly in Italy, where benzylpenicillin resistance is considered a sentinel marker for community-acquired respiratory pathogens.

#### 3.3.2. Ambulatory Patients

*P. aeruginosa* (CRE): Resistance remained low pre-COVID and during COVID (0% and 2.6%, respectively) but surged significantly to 23.5% (12/51) post-COVID (*p* = 0.0014). This sharp increase raises concerns regarding the spread of multidrug-resistant strains in community settings.

*S. aureus* (MRSA): the proportion of MRSA isolates decreased over time (from 15 pre-COVID to 11 during COVID and 2 post-COVID), but the reduction was not statistically significant.

*K. pneumoniae* (CRE): CRE strains were found in 2/9 isolates pre-COVID but were absent during the COVID and post-COVID periods. Although suggestive of a decline, the numbers were insufficient for robust statistical analysis.

*S. pneumoniae* (benzylpenicillin resistance): In ambulatory patients, 2/2 isolates during COVID were resistant, representing a transient 100% resistance rate, which decreased post-COVID. Despite small numbers, this trend aligns with inpatient observations.

*S. pyogenes* (macrolide resistance): Resistance rose from 5.4% (2/37) pre-COVID to 18.2% (2/11) during COVID and slightly decreased to 14.8% (4/27) post-COVID. While not statistically significant, this trend mirrors that of *S. pneumoniae*, suggesting pandemic-related shifts in antibiotic selection pressure.

These findings emphasize how the COVID-19 pandemic influenced antimicrobial resistance dynamics in both hospital and community settings. The emergence of resistant Gram-negative pathogens post-pandemic—especially in outpatients—warrants vigilant surveillance and targeted antimicrobial strategies.

## 4. Discussion

This retrospective study offers a comprehensive overview of bacterial respiratory infection patterns and antimicrobial resistance (AMR) across pre-, during-, and post-COVID-19 periods, stratified by inpatient and outpatient settings. Several key trends emerged from the data: (1) a marked increase in nosocomial pathogens during the COVID-19 period; (2) a higher frequency of polymicrobial infections among outpatients; and (3) selective but significant shifts in resistance, particularly involving carbapenem-resistant *P. aeruginosa* and benzylpenicillin-resistant *S. pneumonia*. Our findings align closely with recent European surveillance data (ECDC, ISS), which reported similar persistent AMR trends in carbapenem-resistant pathogens post-pandemic, reinforcing the need for sustained antimicrobial stewardship.

### 4.1. Impact of the Pandemic on Pathogen Distribution

In hospitalized patients, a significant surge in *P. aeruginosa* and the *A. baumannii complex* was observed during the COVID period. These findings are consistent with previously reported increases in opportunistic pathogens during surges in ICU admissions and ventilator use (5, 7). The rise in *A. baumannii*—from 3 isolates pre-COVID to 59 during COVID (*p* < 0.0001) mirrors its known propensity for causing ventilator-associated pneumonia (VAP) and thriving in high-dependency units. The subsequent decline post-COVID suggests that healthcare system normalization and reestablished infection control protocols may have effectively curtailed its spread [16].

Among ambulatory patients, pathogen distribution was largely influenced by anatomical site. *S. pyogenes* and *H. influenzae* dominated upper respiratory tract infections, while *P. aeruginosa* and *Candida* spp. were more prevalent in lower tract specimens. The significant reduction in *H. influenzae* during the COVID period (*p* = 0.000002) likely reflects the efficacy of non-pharmaceutical interventions such as masking and social distancing, which disproportionately affected droplet-transmitted infections [1].

### 4.2. Polymicrobial Infections: A Distinct Outpatient Burden

Polymicrobial infections were significantly more frequent in ambulatory patients than in inpatients across all periods. This may reflect differences in specimen types (e.g., sputum vs. BAL), patient comorbidities, or sampling methods. Particularly noteworthy is the high rate of *Candida* spp. co-isolation predominantly with bacterial pathogens such as *P. aeruginosa*, *S. aureus*, or *K. pneumoniae* in polymicrobial infections (>70% in lower respiratory samples during pre- and COVID periods). Although traditionally regarded as a colonizer, recent literature supports its potential role as a surrogate marker for dysbiosis and broad-spectrum antibiotic exposure [14,15]. Whether *Candida* contributes to pathogenic synergy in polymicrobial respiratory infections remains an area for further investigation.

### 4.3. Antimicrobial Resistance Trends: Mixed Signals

Several AMR trends identified in this study are clinically relevant. In hospitalized patients, a significant decline in carbapenem-resistant *P. aeruginosa* was observed from pre-COVID (32.1%) to COVID (20.2%) (*p* = 0.0163). This aligns with findings from other settings, where stewardship efforts and infection prevention measures intensified during the pandemic [17]. Similarly, in outpatients, CRPA resistance in *P. aeruginosa* decreased significantly from COVID to post-COVID (*p* = 0.0014).

In contrast, the significant increase in CRE-producing *K. pneumoniae* among outpatients (*p* = 0.0098) during COVID, despite low isolate numbers, raises concerns about community dissemination or hospital discharge-associated transmission. This trend warrants closer longitudinal monitoring.

Of special note is the increase in benzylpenicillin resistance among *S. pneumoniae* during COVID. While not statistically significant due to limited isolate numbers, this finding holds epidemiological importance. In Italy and across Europe, *S. pneumoniae* is a sentinel organism in AMR surveillance, and rising beta-lactam resistance may indicate early warning signs of emerging resistance [2,18].

In *S. pyogenes*, a gradual rise in macrolide resistance was observed across the three periods in outpatients, peaking at 14.8% post-COVID. Although the trend did not reach statistical significance, this upward trajectory—mirrored by similar findings in *S. pneumoniae*—suggests a possible shift in community-level antibiotic exposure, likely linked to the resumption of social interactions and outpatient antibiotic use.

### 4.4. Clinical and Epidemiological Implications

This study confirms the profound effect of the COVID-19 pandemic on respiratory pathogen ecology and resistance profiles. The rise in hospital-acquired, drug-resistant organisms during the pandemic, followed by partial normalization post-COVID, emphasizes the importance of infection prevention continuity even during healthcare crises. Meanwhile, persistent outpatient trends—particularly polymicrobial infections and shifts in *S. pneumoniae* resistance—highlight the need for targeted surveillance outside hospital settings.

Future studies should incorporate molecular typing and resistance gene profiling to explore the mechanisms behind these shifts. Additionally, the consistent detection of *Candida* spp. in polymicrobial infections invites investigation into fungal–bacterial interactions in the respiratory tract. The slight post-COVID increase in *Candida* spp. isolation in outpatients may also reflect the broader return to social interaction and person-to-person contact, which facilitates the transmission of colonizing fungal species, particularly in urban and community settings.

## 5. Conclusions

This study provides a detailed, six-year epidemiological and microbiological analysis of respiratory tract infections (RTIs), highlighting significant shifts in pathogen distribution and antimicrobial resistance (AMR) patterns across the pre-, during-, and post-COVID-19 periods. The pandemic period was marked by a notable surge in hospital-associated pathogens, such as *P. aeruginosa* and *A. baumannii*, reflecting increased ICU burden, mechanical ventilation, and empirical antibiotic use. While many trends began to normalize post-pandemic, the persistence of some resistance phenotypes—particularly in carbapenem-resistant *K. pneumoniae* and *P. aeruginosa*—underscores the lasting impact of pandemic-related healthcare disruptions.

Among the findings of this study, the frequent detection of *Candida* spp. in outpatient respiratory samples—particularly from lower respiratory tract specimens and often within polymicrobial contexts—deserves attention. Although *Candida* is traditionally viewed as a colonizer, its consistent isolation across pandemic phases suggests a possible role as an indirect marker of antimicrobial pressure, dysbiosis, or ecological imbalance in community settings.

Several overlapping factors may account for this pattern. A plausible explanation involves the post-pandemic resumption of social interactions and interpersonal contact, which may have facilitated the transmission of colonizing fungal species among individuals. In parallel, all outpatient samples originated from the Milan metropolitan area, a region characterized by chronic air pollution, including elevated levels of PM_2.5_, NO_2_, and ozone. Environmental exposure to such pollutants has been associated with impaired mucosal immunity and fungal overgrowth in both experimental and clinical studies. Katsoulis et al. [19] demonstrated that PM_2.5_ exposure in murine models facilitated *Candida* albicans lung infection via epithelial barrier disruption. Similarly, Correa-Moreira et al. [20] and Hassanpour et al. [21] reported airway dysbiosis and elevated *Candida* burdens in polluted settings.

While *Candida* spp. was not the only relevant observation in this dataset, its presence—unique to outpatients and persistent across all timeframes—raises important questions about the interplay between the environment, behavior, and microbiota. Further research integrating environmental data, respiratory microbiome profiling, and host immune response will be essential to clarify whether *Candida* spp. functions as a passive bystander or a sentinel organism for pollution-associated respiratory imbalance.

Importantly, the increase in benzylpenicillin resistance in *Streptococcus pneumoniae* during the COVID period, though based on small numbers, signals a potentially emerging resistance trend. Given its role as a sentinel organism in AMR surveillance, this finding merit continued monitoring in both community and hospital settings.

These findings collectively emphasize the importance of sustained, site-specific microbiological surveillance and antimicrobial stewardship. Understanding how public health emergencies alter pathogen dynamics is critical for developing resilient infection control frameworks. Future work should focus on integrating genomic surveillance, evaluating patient outcomes, and exploring the ecological interactions—bacterial and fungal—that shape respiratory infections in both acute and post-crisis healthcare environments.

## 6. Limitations

A limitation of our study is the lack of molecular characterization for *Klebsiella pneumoniae* isolates, which prevents differentiation between hypervirulent and classical strains. Future research should include molecular typing to address this important epidemiological aspect. The correlation between bacterial respiratory infections and the SARS-CoV-2 positivity status was not assessed in this study, representing another limitation and an area for future investigation. Additionally, as this was a retrospective study based on the totality of available respiratory samples, no formal sample size calculation was performed, which may limit the power of subgroup analyses

## Figures and Tables

**Table 1:** Distribution of bacterial isolates across the three study periods.

Period	Number of Isolates
Pre-COVID	593
COVID	796
Post-COVID	438
Total	1827

**Table 2:** Distribution of major respiratory bacterial pathogens isolated from hospitalized patients across the three study periods: pre-COVID (June 2018–December 2019), COVID (January 2020–July 2022), and post-COVID (August 2022–June 2024).

Bacteria Isolates	Pre-COVID	COVID	Post COVID
*Acinetobacter baumannii complex*	3	59	8
*Hemophilus influenzae*	23	9	20
*Klebsiella pneumoniae*	45	100	39
*Pseudomonas aeruginosa*	112	248	150
*Staphylococcus aureus*	58	108	35
*Streptococcus pneumoniae*	4	5	4
Total	246	530	256

**Table 3:** Anatomical distribution of respiratory pathogens isolated from ambulatory patients across the three study periods.

Bacteria Isolates	Pre-COVID		COVID		Post-COVID	
	Low Respiratory Tract	Upper Respiratory Tract	Low Respiratory Tract	Upper Respiratory Tract	Low Respiratory Tract	Upper Respiratory Tract
*Candida* spp.	61	44	48	40	11	38
*Hemophilus influenzae*	32	21	23	0	21	4
*Klebsiella pneumoniae*	9	0	7	0	3	0
*Pseudomonas aeruginosa*	36	14	72	7	51	12
*Staphylococcus aureus*	21	63	15	40	4	9
*Streptococcus pneumoniae*	0	4	1	1	2	0
*Streptococcus pyogenes*	0	37	0	11	0	27
Total	347		266		182	

## Data Availability

The data that support the findings of this study are not openly available due to reasons of sensitivity and are available from the corresponding author upon reasonable request. Data are located in controlled access data storage at Multimedica IRCCS.

## Supplementary Materials

The following supporting information can be downloaded at https://www.mdpi.com/article/10.3390/antibiotics14080796/s1, Figure S1: Heatmap illustrating the percentage of antibiotic-resistant respiratory pathogens isolated from hospitalized patients across the pre-COVID (June 2018–December 2019), COVID (January 2020–July 2022), and post-COVID (August 2022–June 2024) periods; Figure S2: Heatmap of Respiratory Microorganism Isolates by Patient Setting and Pandemic Period; Table S1: Distribution of respiratory pathogens isolated from ambulatory patients by anatomical site and pandemic period; Table S2: Detailed distribution of respiratory pathogens isolated from ambulatory patients by sample type and pandemic period; Table S3: Distribution of antimicrobial-resistant respiratory bacterial isolates across pre-COVID (June 2018–December 2019), COVID (January 2020–July 2022), and post-COVID (August 2022–June 2024) periods.
