# Supplementary material for: Evolution of Respiratory Pathogens and Antimicrobial Resistance over the COVID-19 Timeline: A Study of Hospitalized and Ambulatory Patient Populations
Source: Antibiotics (Basel). 2025 Aug 5;14(8):796. doi: 10.3390/antibiotics14080796 (PMC12382871; doi:10.3390/antibiotics14080796)
Supplement: Supplementary file 1 [file antibiotics-14-00796-s001.zip › antibiotics-3794359-supplementary.pdf]

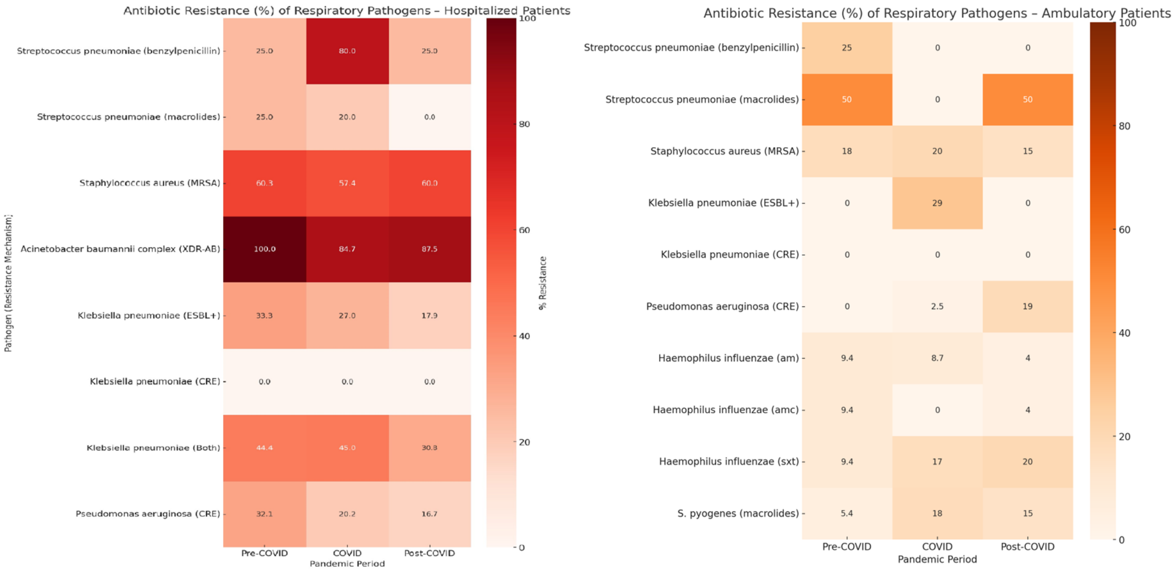

Figure S1: Heatmap illustrating the percentage of antibiotic-resistant respiratory pathogens isolated from hospitalized patients across the pre-COVID (June 2018–December 2019), COVID (January 2020–July 2022), and post-COVID (August 2022–June 2024) periods. Resistance profiles include MRSA (Staphylococcus aureus), XDR-AB (Acinetobacter baumannii complex), ESBL+ and/or CRE (Klebsiella pneumoniae), CRPA (Pseudomonas aeruginosa), and resistance to benzylpenicillin and/or macrolides in Streptococcus pneumoniae. Percentages were calculated by normalizing the number of resistant isolates to the total number of isolates per species and period. Only pathogens with at least one isolate per period were included. Notably, A. baumannii complex exhibited 100% XDR resistance in all periods, while S. pneumoniae showed a spike in benzylpenicillin resistance during the COVID period.

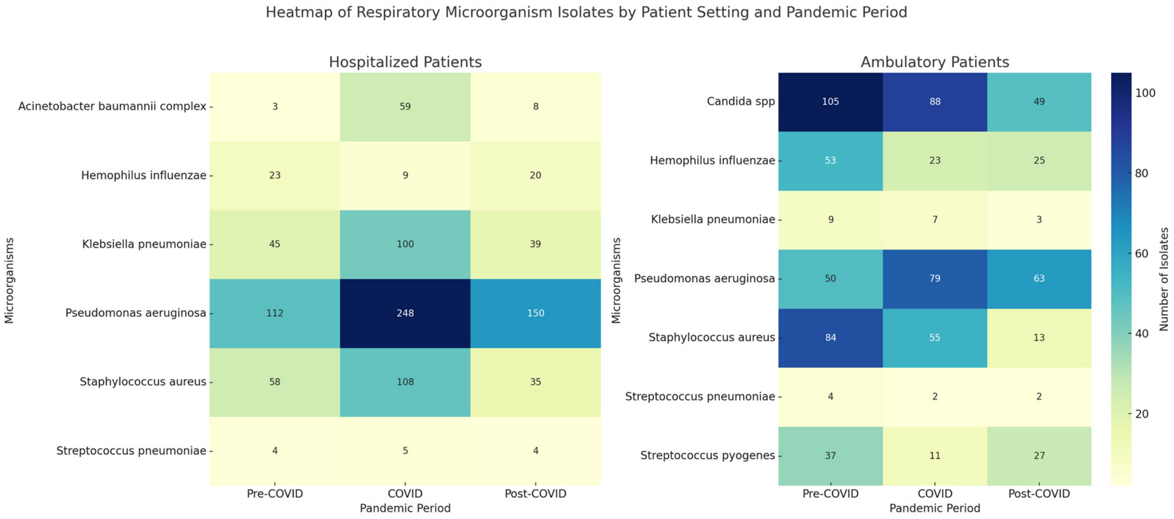

Figure S2: Heatmap of Respiratory Microorganism Isolates by Patient Setting and Pandemic Period: This figure presents the distribution of respiratory microbial isolates collected from hospitalized (left panel) and ambulatory (right panel) patients across three defined pandemic periods: pre-COVID (June 2018–December 2019), COVID (January 2020–July 2022), and post-COVID (August 2022–June 2024). Each cell indicates the absolute number of isolates identified for a given pathogen during each period. In hospitalized patients, Pseudomonas aeruginosa and Klebsiella pneumoniae were dominant pathogens, with a marked surge in Acinetobacter baumannii complex during the COVID phase. Among ambulatory patients, Candida spp. was frequently isolated across all periods, while Streptococcus pyogenes and Staphylococcus aureus predominated in the pre-COVID and post-COVID periods. The heatmap highlights distinct pathogen distribution patterns between inpatient and outpatient settings, as well as temporal shifts potentially linked to healthcare dynamics and community transmission trends.

Supplementary Tables:

Table S1: Distribution of respiratory pathogens isolated from ambulatory patients by anatomical site and pandemic period. *The table presents absolute counts of isolates from lower and upper respiratory tract samples across three timeframes: pre-COVID (June 2018–December 2019), COVID (January 2020–July 2022), and post-COVID (August 2022–June 2024).*

Bacteria isolates	Pre COVID		COVID		post COVID	
	BAS/BAL	ESP	BAS/BAL	ESP	BAS/BAL	ESP
Acinetobacter baumannii complex	2	1	52	7	5	3
Hemophilus influenzae	15	8	5	4	15	5
Klebsiella pneumoniae	41	4	88	12	34	5
Pseudomonas aeruginosa	88	24	199	49	112	38
Staphylococcus aureus	47	11	92	16	24	11
Streptococcus pneumoniae	4	0	4	1	3	1
Moraxella catarrhalis	1	0	1	0	0	0
**Total**	198	48	441	89	193	63
**Total isolate bacteria**	246		530		256	

Table S2: Detailed distribution of respiratory pathogens isolated from ambulatory patients by sample type and pandemic period. *The table reports the absolute number of isolates stratified by anatomical sampling site: bronchoalveolar lavage/bronchoaspirate (BAL/BAS), sputum (ESP), tonsillar/pharyngeal swabs (COLTFAR), tracheal aspirates (TAU), and nasal swabs (TNAS), across three pandemic phases: pre-COVID (June 2018–December 2019), COVID (January 2020–July 2022), and post-COVID (August 2022–June 2024).*

Bacteria isolates	Pre COVID					COVID					post COVID				
	BAS/BAL	ESP	COLTFAR	TAU	TNAS	BAS/BAL	ESP	COLTFAR	TAU	TNAS	BAS/BAL	ESP	COLTFAR	TAU	TNAS
Candida spp	32	29	33	11	0	29	19	33	5	2	5	6	33	3	2
Hemophilus influenzae	20	12	0	2	19	10	13	0	0	0	13	8	0	0	4
Klebsiella pneumoniae	5	4	0	0	0	3	4	0	0	0	2	1	0	0	0
Pseudomonas aeruginosa	21	15	0	12	2	36	36	0	3	4	26	25	0	10	2
Staphylococcus aureus	12	9	0	11	52	11	4	0	13	27	4	0	0	6	3
Streptococcus pneumoniae	0	0	0	0	4	1	0	0	0	1	2	0	0	0	0
Streptococcus pyogenes	0	0	37	0	0	0	0	11	0	0	0	0	27	0	0
Moraxella catarrhalis	0	1	0	0	4	0	1	0	0	0	0	0	0	0	0
**Total**	90	70	70	36	81	90	77	44	21	34	52	40	60	19	11

**Total respiratory bacteria**	347	266	182

*Table S3: Distribution of antimicrobial-resistant respiratory bacterial isolates across pre-COVID (June 2018–December 2019), COVID (January 2020–July 2022), and post-COVID (August 2022–June 2024) periods. The table reports the number of resistant isolates for selected respiratory pathogens stratified by care setting (hospitalized [INT] vs. ambulatory [EXT]) and resistance phenotype. Resistance mechanisms include MRSA (*Staphylococcus aureus*), XDR-AB (*Acinetobacter baumannii*), CRPA (*Pseudomonas aeruginosa, Klebsiella pneumoniae*), ESBL+ (*K. pneumoniae*), and resistance to benzylpenicillin and/or macrolides in* Streptococcus pneumoniae *and* Streptococcus pyogenes. *Resistance phenotypes for* Haemophilus influenzae *include ampicillin (am), amoxicillin/clavulanic acid (amc), and trimethoprim-sulfamethoxazole (sxt).*

Isolate bacteria	Pre COVID			COVID			post COVID		
Streptococcus pneumoniae INT	Benzylpenicillin	Macrolides	Both	Benzylpenicillin	Macrolides	Both	Benzylpenicillin	Macrolides	Both
	1	1	0	0	0	4	1	1	0
Staphylococcus aureus INT	MRSA			MRSA			MRSA		
	35			62			21		
Acinetobacter baumannii complex INT	XDR-AB			XDR-AB			XDR-AB		
	3			50			7		
Staphylococcus aureus EXT	MRSA			MRSA			MRSA		
	15			11			2		
Streptococcus pneumoniae EXT	Benzylpenicillin	Macrolides	Both	Benzylpenicillin	Macrolides	Both	Benzylpenicillin	Macrolides	Both
	1	2	1	0	0	0	0	1	0
Klebsiella pneumoniae INT	ESBL+	CRE	Both	ESBL+	CRE	Both	ESBL+	CRE	Both
	15	0	20	27	0	45	7	0	12
Klebsiella pneumoniae EXT	ESBL+	CRE	Both	ESBL+	CRE	Both	ESBL+	CRE	Both
	0	0	2	2	0	0	0	0	0
Pseudomonas aeurginosa INT	CRPA			CRPA			CRPA		
	36			50			25		
Pseudomonas aeurginosa EXT	CRPA			CRPA			CRPA		
	0			2			12		
Hemophilus influenzae INT	AM	AMC	SXT	AM	AMC	SXT	AM	AMC	SXT
	3	2	4	0	0	2	1	0	2
Hemophilus influenzae EXT	AM	AMC	SXT	AM	AMC	SXT	AM	AMC	SXT
	5	5	5	2	0	4	1	1	5
S.pyogenes EXT	Macrolides			Macrolides			Macrolides		
	2			2			4		
